# Biological functions of endophytic bacteria in *Robinia pseudoacacia* ‘Hongsen*’*

**DOI:** 10.3389/fmicb.2023.1128727

**Published:** 2023-08-09

**Authors:** Minqing Huang, Lijing Chen, Jiasi Ma, Jingzhi Mo, Lu He, Qihua Liang, Guixiang Peng, Zhiyuan Tan

**Affiliations:** ^1^College of Agriculture, South China Agricultural University, Guangzhou, China; ^2^College of Natural Resources and Environment, South China Agricultural University, Guangzhou, China

**Keywords:** *Robinia pseudoacacia* ‘Hongsen’, endophytic bacteria, rhizosphere soil bacteria, high-throughput sequencing, biodiversity and biological function

## Abstract

**Introduction:**

Endophytes and their host plants have co-evolved for a very long time. This relationship has led to the general recognition of endophytes as a particular class of microbial resources. *R. pseudoacacia* ‘Hongsen’ is drought- and barren-resistant species that can be grown in both the north and south of China, efficiently addresses the ecological issues caused by China’s ‘southern eucalyptus and northern poplar. Up to date, cultured-dependent studies are available for the *R. pseudoacacia* nitrogen-fixing and other endophytes. Therefore, the present research studied the *R. pseudoacacia* ‘Hongsen,’ microbiome in detail by high-throughput sequencing and culture dependant.

**Methods:**

This study examined microbial species and functional diversity in *Robinia pseudoacacia* ‘Hongsen’ using culture-dependent (isolation) and culture-independent techniques.

**Results:**

A total of 210 isolates were isolated from *R. pseudoacacia* ‘Hongsen.’ These isolates were clustered into 16 groups by the In Situ PCR (IS-PCR) fingerprinting patterns. 16S rRNA gene sequence analysis of the representative strain of each group revealed that these groups belonged to 16 species of 8 genera, demonstrating the diversity of endophytes in *R. pseudoacacia* ‘Hongsen’. ’*Bacillus* is the most prevalent genus among all the endophytic bacteria. High-throughput sequencing of endophytic bacteria from *R. pseudoacacia* ‘Hongsen’ of the plant and the rhizosphere soil bacteria showed that the bacterial populations of soil near the root, leaf, and rhizosphere differed significantly. The microbial abundance decreased in the endophytes as compared to the rhizosphere. We observed a similar community structure of roots and leaves. With and without root nodules, *Mesorhizobium* sp. was significantly different in *R. pseudoacacia* ‘Hongsen’ plant.

**Discussion:**

It was predicted that *R. pseudoacacia* ‘Hongsen’ plant endophytic bacteria would play a significant role in the metabolic process, such as carbohydrate metabolism, amino acid metabolism, membrane transport, and energy metabolism.

## Introduction

*Robinia pseudoacacia* ‘Hongsen’, a deciduous tree belonging to the genus *Robinia* in the family Fabaceae, has a well-developed root system with nodules, a straight trunk, a complete crown, evenly distributed branches, tender and soft leaves, pure white and fragrant flowers, and is an excellent fast-growing variety (Hu et al., 2021). Being a drought- and barren-resistant species that can be grown in both the north and south of China, *R. pseudoacacia* ‘Hongsen’ efficiently addresses the ecological issues caused by China’s ‘southern eucalyptus and northern poplar. It has a wide range of application prospects. However, further study is needed on the endophytic bacteria of *R. pseudoacacia* ‘Hongsen’. Up to date, cultured-dependent studies are available for the *R. pseudoacacia* nitrogen-fixing and other endophytes. Therefore, the present research studied the *R. pseudoacacia* ‘Hongsen’, microbiome in detail by high-throughput sequencing. Plant endophytes are microorganisms like bacteria and fungi that colonize healthy plant tissues without causing any adverse effects and maintain a stable symbiotic relationship by secreting a variety of bioactive compounds that encourage plant growth (Nair and Padmavathy, 2014). Plant endophytes mainly include three categories: endophytic bacteria, endophytic fungi, and endophytic actinomycetes (Afzal et al., 2019). Its sources can be loosely separated into horizontal transmission channels of soil-invading strains and bacteria in the environment through aerial tissues and flower organs of leaves and vertical transmission paths of strains through vertical migration of seeds and vertical distribution of pollen (Long et al., 2013; Hassani et al., 2018). Freeman isolated the first plant endophytes from *Lolium temulentum* in 1904 (Kusari et al., 2012). Technological advancement and continuous study of plant endophytes have made significant progress with their application value in microbial community research. Numerous types of plant endophytes exist throughout the plant’s tissues, and have been studied in different plants like rice (*Oryza sativa*), corn (*Zea mays*), sugar beet (*Beta vulgaris*), eucalyptus (*Eucalyptus urophyll*), pine (*Pinus seylvestris*), herbs, and algae (Wagner et al., 2016). More than 100 bacterial species, predominated by *Enterobacter*, *Pseudomonas*, and *Bacillus* are associated with plants as endophytic bacteria (Stone et al., 2000; Gangadevi and Muthumary, 2009).

Harmful microbes are the cause of biotic stress that prevents plant’s normal growth along with various negative effects on global crop production. The main biological stress factors (BSF) like virus, bacteria, fungi, nematods, insects, and weeds are responsible for the plants elevated reactive oxygen species (ROS) that negatively affect the plant physiology and the cause of alleviated crop productivity (Gouda et al., 2016; Huang et al., 2019). The environmental friendly bacterial and fungal endophytes are the solution to the problems that faced during conventional forming as they colonize in plant tissues without any pathological issues (Chaudhary et al., 2022). Endophytes not only help the host for their nutrients, but also diminish the injury of plant tissues caused by phytopathogen via antibiotics, lytic enzymes, certain hormones activation, and secondary metabolites. Therefore, endophytes are considered the future biofertilizers and biocontrol agent due to eco-friendly nature.

The research methods of microbial diversity are primarily classified into culture-dependent and culture-independent (Lasken, 2012; Dodsworth et al., 2013). Traditionally endophytes were isolated by grounding up plant tissues or organs. Selective medium, culturing, and sub-culturing were used to isolate pure strains, which were then subject to morphological, physic-chemical, and functional analysis. However, not all endophytes can be cultured *In Situ* due to several limitations. These are the insensitivity of detection technology to microorganisms with low growth rates in oligotrophic environments (Simu and Hagström, 2004), eutrophication of the medium matrix, ignorance of the interactions between microorganisms (Singh et al., 2000), and the inability to replicate the original ecological environment (Sanmee et al., 2010; Trevors, 2011; Jon McIlroy et al., 2013). Comparing the bacteria isolated by traditional culture methods with the Global Bacterial Diversity Index found that the number that cultured by traditional cultural method was less than 1.0% of the total (Salam et al., 2020), while prokaryotes were only 0.1% (Alain and Querellou, 2009).

Culture-independent methods include denaturant gradient gel electrophoresis (DGGE), real-time RT-PCR, fluorescence *in situ* hybridization (FISH), and high-throughput sequencing (Li et al., 2020). Among them, high-throughput sequencing technology has seen a sharp rise with the concept of the metagenome because it has three benefits: sequencing throughput, a high reflection of sample species and the number of species, high sequencing rate, and low cost. It has since become a popular method of microbial diversity research (Ji et al., 2020). Microbial species, community, functional, and genetic diversity can be studied using high-throughput sequencing technologies (Stepanauskas, 2012). Li and co-workers utilized high-throughput sequencing technology (HTST) to demonstrate that continual planting drastically altered soil and endophytic bacteria’s community structure in sugar beet (Li et al., 2020). Similarly, the HTST study revealed that the microbial community genes related to the carbon and nitrogen cycle changed rapidly after thawing permafrost (Mackelprang et al., 2011).

There is a dearth of domestic and international research on the endophytic bacteria of *R. pseudoacacia* ‘Hongsen’. However, *R. pseudoacacia* ‘Hongsen’ endophytic bacteria and its soil bacterial diversity based on high-throughput sequencing technology need to be studied. Therefore, the current report studied the bacterial diversity (rhizosphere and endosphere), abundance, structure, and composition in various compartments of *R. pseudoacacia* ‘Hongsen’. In addition, the study also predicted the functions and different metabolic pathways of the bacterial community associated with the studied plant.

## Materials and methods

### Isolation, purification, preservation of endophytes

*Robinia pseudoacacia* ‘Hongsen’ plants provided by Anhui Hongsen Hi-Tech Forestry Co., Ltd., planted in Shaoguan Planting Base in Guangdong Province (24°56′51″N, 114°05′52″E; 119.7 m). A total of 5 plants were used in this experiment. The leaves, roots, and nodules of the collected *R. pseudoacacia* ‘Hongsen’ plants were washed with sterile water. The different plant tissues were cut into pieces of about 3 cm, rinsed with sterile water several times in succession, and surface sterilized with 75% ethanol solution for 5 min, then rinsed with sterile water for five times to remove the residual ethanol. For sterility test, the plant material were placed in LB solid medium and incubated for 2 days at 37°C. No microbial growth of medium surface showed the plant materials are surface sterilized. Plant tissues that surface sterilized were placed into an autoclaved mortar and placed with a tweezer. Sterile water was continuously added during the grinding process and diluted the concentration gradient to 10^−3^, 10^−4^, and 10^−5^. poured 50 μL of dilutions with different concentration gradients on different solid media such as LB, BPA (Guo et al., 2011), YPD (Chauhan and Kruppa, 2009), KMB (Kourtchenko et al., 2018), NA (Barrios-González, 2012), DN (Barrios-González, 2012). The Petri plates were then incubated at 30°C for 5 to 8 days. Observing the solid media every 8 h, choose, record, and take pictures of the bacterial single colony. Colonies were separated by a sterile wire loop and streak plate method was used to sub-culture them. The pure culture was preserved in a solution containing 20.0 mL of glycerol, 1.5 g of skim milk powder, and 6 mL of 0.5% bromophenol blue solution per 100 mL. Mixed well the bacteria with the solution and stored at −80°C.

### IS-PCR fingerprinting

The IS-PCR fingerprinting technology was used to conduct a molecular-level cluster analysis of strains. The DNA of each strain was used as a template, and J3(5′-GCT CAG GTC AGG TGG CCT GG-3′) single primers were used for PCR amplification. The PCR reaction mixture (25 μL total volume) consisted of: 2 × Taq PCR Mix 12.5 μL, 1.0 μL primer (50.0 μmolL^−1^), template DNA (40.0 ng μL^−1^) 0.5 μL, ddH2O 11.0 μL. Moreover, PCR reaction conditions are 95°C for 5 min; then 95°C for 50 s, 56°C for 50 s, 72°C for 1 min, 35 cycles; Finally 72°C for 5 min. Following 1% agarose gel electrophoresis to identify the PCR product, 6% polyacrylamide gel electrophoresis (PAGE) was used to separate the bands; after the separation, the GIS UV gel imaging system was used to take pictures and analyze the clustering results.

### 16S *rRNA* gene sequence analysis of the isolates

The 16S *rRNA* gene was amplified using primers 27F (5′-AGA GTT TGA TCC TGG CTC AG-3′) and 1492R (5′-GGT TAC CTT GTT ACG ACT T-3′), then sequenced using the ABI PRISM ® 3730 Genetic ABI PRISM® 3730 Genetic Analyzer (Applied Biosystems, Foster City, CA, USA) with the BigDye ® Terminator v3.1 Cycle Sequencing Kit (Applied Biosystems) being used for sequencing reactions. To find the 16S *rRNA* gene sequences with the highest degree of similarity, the obtained sequences were compared with the NCBI database in EzBioCloud (Yoon et al., 2017). ClusterW was then used for multiple sequence alignment, and MEGA 11 (Tamura et al., 2021) was used to construct the phylogenetic tree by proximity method and determine its phylogenetic status.

### Strain physiological and biochemical tests

Each isolate was subject to methyl red, acetyl methyl methanol, ammonia production, urease, gelatin liquefaction, catalase, and nitrate reduction test.

### Biological function determination of strains

There are currently various techniques to determine strain’s biological function.

### P solubilization

We followed the method of Li et al. (2019) for P solubilization of bacterial strains.

In the qualitative protease assay, various representative strains were grown on nutrient agar plates containing (1%) casein as substrate. Clear zones of hydrolysis around the colonies confirmed the proteolytic activity of the strain (Afzal and Ihsan-Ud-din, 2021). To check the cellulose activity, selected strains were grown on Carboxymethylcellulose (CMC) agar media. To visualize the hydrolysis zone, the plates were flooded with an aqueous solution of 0.1% Congo red for 15 min and washed with 1 M NaCl (Sethi et al., 2013). The Salkowski (Glickmann and Dessaux, 1995) method was used to measure the quantity of auxin in the culture medium. All the representative strains were activated in an LB liquid medium to determine siderophore capacity; after centrifugation, pipetted 1.0 mL bacterial suspension into a tube that contained 5.0 mL MSA-CAS liquid medium (Song et al., 2021). Each representative strain was then inoculated in three tubes. Finally checked, the color of the medium after 72 h kept on shaking at 150 rpm at 37°C.

### High-throughput sequencing to analyze endophytic bacteria and rhizosphere soil bacterial diversity

#### Material collection and processing

*Robinia pseudoacacia* ‘Hongsen’ and its rhizosphere soil were collected from Shaoguan Planting Base in Guangdong Province (24°56′51″N, 114°05′52″E; 119.7 m) in China. The soil samples were air-dried and sieved to pass a 2-mm sieve to determine baseline soil properties (Afzal et al., 2022). The description of the samples is shown in Table 1.

**Table 1:** The sample name.

	*R. pseudoacacia* ‘Hongsen’ (Root contains rhizomes)	*R. pseudoacacia* ‘Hongsen’ (The root does not contain the nodules)
Roots/roots and rhizomes	YG	WG
Leaf	YY	WY
Rhizosphere soil	YT	WT

In this study, we follow the definition of rhizosphere as the soil attached to roots at a distance of approximately 1 mm (Edwards et al., 2015). To collect rhizosphere soil, the root system was transferred to Falcon tubes containing 50 mL of sterile phosphate-buffered saline (PBS) solution. The roots were sonicated to remove from the tubes, and the tubes were centrifuged to collect the soil samples. The endophytic compartment was isolated following the previous protocol (Lebeis et al., 2015). Briefly, the roots were placed in the sterile distilled water and rinsed for 2–3 times and the debris was removed aseptically. Sonication was performed for 30 s at 50–60 Hz (output frequency 42 kHz, power 90 W, Branson Ultrasonics) and treated with 80% ethanol for 2 min. All centrifuged fresh samples from the rhizosphere and sonicated roots endosphere were stored at −80°C until microbial DNA isolation.

#### Leaf sample

Fresh shoot samples were collected and rinsed with sterile water to remove the surface dust and microbes, and soaked the leavesin 80% ethanol solution for 2 min. Then put in 1% NaClO solution for 5 min. After soaking, they were finally disinfected in 80% ethanol solution for 1 min and washed seven times with sterile water shaking. The cleaned samples were stored in 2 mL sterile EP tubes in three replicates at −80°C till sequencing.

#### Total DNA extraction and purification

The TGuide S96 magnetic bead soil/fecal genomic DNA extraction kit (Azimi et al., 2011) was used to extract the bacterial DNA, and the product was detected using 1.0% agarose gel electrophoresis. Clear band samples and > 10 ng/L DNA samples were selected for further analysis.

### Library construction and sequencing

For PCR, a concentration of 1 ng μL^−1^ of template DNA was used to amplify 16S *rRNA* gene V3-V4 region with primers 335F (5′-CAD ACT CCT ACG GGA GGC-3′), 769R (5’-ATC CTG TTT GMT MCV CRC-3′), primer 338F (5’-ACT CCT ACG GGA GGC AGC A-3′), and 806R (5′-GGA CTA CHV GGG TWT CTA AT-3′). The conditions of PCR were the same as ‘IS-PCR fingerprinting’ while DNA integrity and size were observed on a 1.8% agarose gel.

Paired-end reads are merged by using FLASH from original DNA fragments (Magoč and Salzberg, 2011). Paired-end reads were assigned to each sample according to the unique barcodes. These sequencing were then clustered at 97% similarity using UPRASE algorithm (Edgar, 2013), yielding operational taxonomy units (OTUs). remove.lineage command was used to remove any sequence similar to chlorophyll and mitochondrial DNA. For taxonomic classification of OTUs, EzBioCloud database was used (Yoon et al., 2017). For each sample, the sequences were rarefied to the depth of 20,000. Then MUSCLE was used to align the representative sequences of each OTU (Edgar, 2004) and phylogenies were constructed using FastTree (Price et al., 2010) to get an approximate maximum likelihood tree. Jointly using VSEARCH (Rognes et al., 2016), USEARCH (Edgar, 2010), and QIIME (Kuczynski et al., 2012) platforms to perform the above analysis.

### Data preprocessing

The functional potentials of the bacterial communities in soil samples were predicted via phylogenetic investigation of the communities by reconstructing the unobserved states pipeline using PICRUSt v1.1.2 (Douglas et al., 2020) and FAPROTAX (Xu et al., 2021). Functional profiling was conducted using the Kyoto Encyclopedia of Genes and Genomes (KEGG) database.

### Statistical analysis

Mothur (version v.1.30) was used to determine the alpha diversity indices, including Shannon diversity (Keylock, 2005), Chao1 (Chao, 1984), Simpson (Keylock, 2005), and Ace (Chao and Li, 1992). R (v. 3.4.2) was used for correlation analysis, data presentation, and statistical analysis of 16S rDNA (Mazucheli et al., 2018). β diversity analysis was performed to examine similarities and differences between different soil groups, including principal co-ordinates analysis (PCoA) (Huffman et al., 2002), unweighted pair group method with arithmetic mean (UPGMA) (Segura-Alabart et al., 2022), and non-metric multidimensional scaling (NMDS) (Gao et al., 2017). PCA evaluated variations in the microbial community across the two treatments. ggplot2 (Kahle and Wickham, 2013) was used to prepare the graphs. These steps were performed using the R platform with the vegan and ggplot2 packages (Kahle and Wickham, 2013). SourceTracker (v.1.0) (Knights et al., 2011) based on the Bayesian approach was used to estimate the sources of the RR, RS, and RI bacterial communities in each compartment.

## Results

### Isolation and purification of endophytic bacteria from

Dilution and streak plate methods were used to isolate bacteria from the root, root nodules, and leaves. A total of 120 endophytes were isolated from different compartments shown in Supplementary Table 2.

Seventy-two strains were identified from leaves, making up 34.29% of the total bacteria. In contrast, 138 strains were obtained from roots and root nodules, making up 65.71% of the total number of bacteria. Roots and nodules contained much more endophytic bacteria than leaves. There is a correlation between the number of endophytic bacteria identified in each medium. The most bacteria were found in the KMB medium, followed by BPA medium, LB medium, NA medium, DN medium, and YPD medium, by percentage it was 23.33, 22.38, 18.10, 15.71, and 7.14% of the total bacteria, respectively.

### Cluster analysis of endophytes

According to the results of polyacrylamide gel electrophoresis, strains with consistent number, size, and brightness of fingerprint bands were clustered into one category (Supplementary Figure S1). The isolated strains were divided into 16 groups, and one strain was chosen as the representative strain for each group. These sample strains are selected for further experiments. The representative strain for each group is shown in Table 2.

**Table 2:** Sequence similarity of 16S rRNA gene of representative strains of *R. pseudoacacia* ‘Hongsen’.

Groups	Representative strains	The model strain with the highest similarity	Similarity
Ι	LG10	*Bacillus zanthoxyli* 1433^T^	99.2%
II	BG6	*Pseudomonas aeruginosa* JCM 5962^T^	98.6%
III	BG21	*Priestia aryabhattai* B8W22^T^	99.0%
IV	BG30	*Bacillus zhangzhouensis* DW5-4^T^	98.5%
V	BY2	*Bacillus velezensis* CR-502^T^	100.0%
VI	YG8	*Salmonella enterica subsp. diarizona*e DSM 14847^T^	98.3%
VII	YY2	*Bacillus tequilensis* KCTC 13622^T^	99.1%
VII	KG3	*Pseudomonas mosselii* CIP 105259^T^	99.9%
IX	KG6	*Bacillus wiedmannii* FSL W8-0169^T^	98.9%
X	KG19	*Bacillus amyloliquefaciens* DSM 7^T^	99.7%
XI	KG39	*Rummeliibacillus pycnus* NBRC 101231^T^	99.2%
XII	KG43	*Shinella fusca* DC-196^T^	99.4%
XIII	QG1	*Bacillus siamensis* KCTC 13613^T^	99.8%
XIV	QG2	*Priestia megaterium* NBRC 15308^T^	99.9%
XV	QY2	*Bacillus pumilus* ATCC 7061^T^	99.9%
XVI	DY5	*Microbacterium paraoxydans* NBRC 103076^T^	98.8%

### Identification and phylogenetic analysis of representative strains

The 16S rRNA gene of the representative strains PCR and sequenced; clean sequences were compared with the NCBI database using the EzBioCloud database to find the strains that shared similar sequencing. Those with maximum similarity in sequence are shown in Table 2.

The representative strains showed diversity (Table 2). These strains shared 98–100% sequence similarity of the 16S *rRNA* gene. The highest similarity is between BY2 of V cluster and *Bacillus velezensis* CR-502^T^ (100.0%); the lowest is YG8 of taxon VI and *Salmonella enterica* subsp. *diarizonae* DSM 14847^T^ (98.3%).

**Figure 1:**
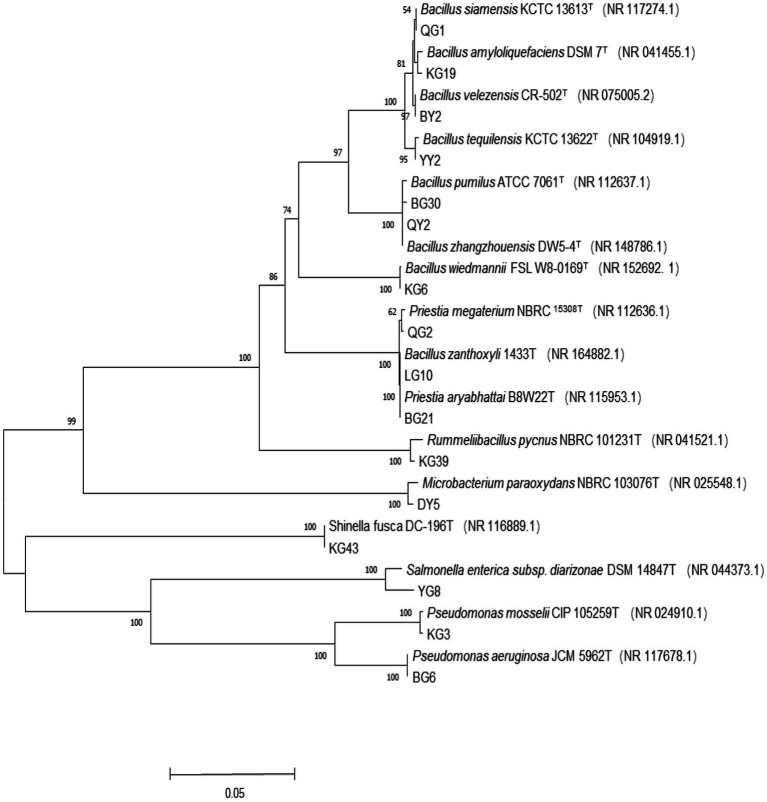
Phylogenetic tree of 16S rRNA gene sequence of *R. pseudoacacia* ‘Hongsen’ and related strains. Tree building method is an adjacency method, T represents the model strain, the ruler represents the nucleotide base difference of 0.05, the branch number represents the self-distance value, the number of random sampling calculations is 1000, and the brackets are the serial number obtained by the model strain Genbank.

The evolutionary history was inferred using the Neighbor-Joining method (Saitou and Nei, 1987) (Figure 1). The percentage of replicate trees in which the associated taxa clustered together in the bootstrap test (1000 replicates) is shown below the branches (Felsenstein, 1985). The tree is drawn to scale, with branch lengths in the same units as those of the evolutionary distances used to infer the phylogenetic tree. The evolutionary distances were computed using the Maximum Composite Likelihood method (Tamura et al., 2004). Evolutionary analyses were conducted in MEGA11 (Tamura et al., 2013). The 16 representative strains of *R. pseudoacacia* ‘Hongsen’ were grouped in the same branch as the strain with the highest similarity, allowing us to infer their phylogenetic position and relationship. According to the 16S rRNA gene phylogenetic analysis*, Bacillus* was the majority genus of endophytes, with 8 of the 16 strains representing the genus, *Priestia* having two representative strains, and the other genera having one representative strain each.

### Physiological and biochemical tests

Most of the isolates were positive for catalase and Ammonia production. In addition, most of the isolates can reduce nitrate (Table 3). It shows that these endophytes could help in the protection of the plants during abiotic and nutrient stresses.

**Table 3:** The physiological and biochemical test of strains.

Strain number	Methyl red test	Acetyl methyl methanol test	Ammonia production test	Urease test	Gelatin liquefaction test	Catalase test	Proteinase production	Cellulase production	Siderophore production	Nitrate reduction test
LG10	+	−	+	+	+	+	−	−	−	−
BG6	+	+	+	+	−	+	+	+	+	+
BG21	+	−	+	+	+	+	+	−	−	−
BG30	+	+	+	−	−	+	+	−	−	−
BY2	−	+	+	−	+	+	+	+	−	+
YG8	+	+	+	+	−	+	−	−	+	+
YY2	+	+	+	+	+	+	+	+	−	+
KG3	+	−	−	+	−	+	−	−	+	+
KG6	−	+	+	−	+	+	+	+	−	+
KG19	−	+	+	−	+	+	+	−	−	−
KG39	−	−	−	+	−	+	−	−	−	−
KG43	−	−	+	−	−	+	−	−	−	+
QG1	−	+	+	−	+	+	+	+	−	+
QG2	+	−	+	+	+	+	+	−	−	+
QY2	+	+	+	−	+	+	+	+	−	−
DY5	−	−	−	−	−	+	+	−	−	−

“+” indicates the test was positive, “−” shows the test was negative.

### Quantitative and qualitative of plant growth-promoting characteristics of isolates

The amount of P and K solubilization has been measured for different strains. Furthermore, protease and cellulase enzyme production were qualitatively detected on Petri plates and test tubes (Table 4).

**Table 4:** Growth-promoting characteristics of strains.

Strain number	Soluble P content (mgL^−1^)	Soluble K content (mgL^−1^)	IAA (mgL^−1^)
LG10	0	0	16.14
BG6	130.82	90.31	0
BG21	0	57.06	4.25
BG30	83.68	33.45	0
BY2	96.03	0	3.80
YG8	117.92	116.59	0
YY2	75.24	0	0
KG3	0	89.90	39.78
KG6	0	0	0
KG19	0	0	0
KG39	0	0	11.98
KG43	0	0	23.34
QG1	36.47	0	0
QG2	0	0	0
QY2	55.00	85.40	0
DY5	0	0	17.28

**Figure 2:**
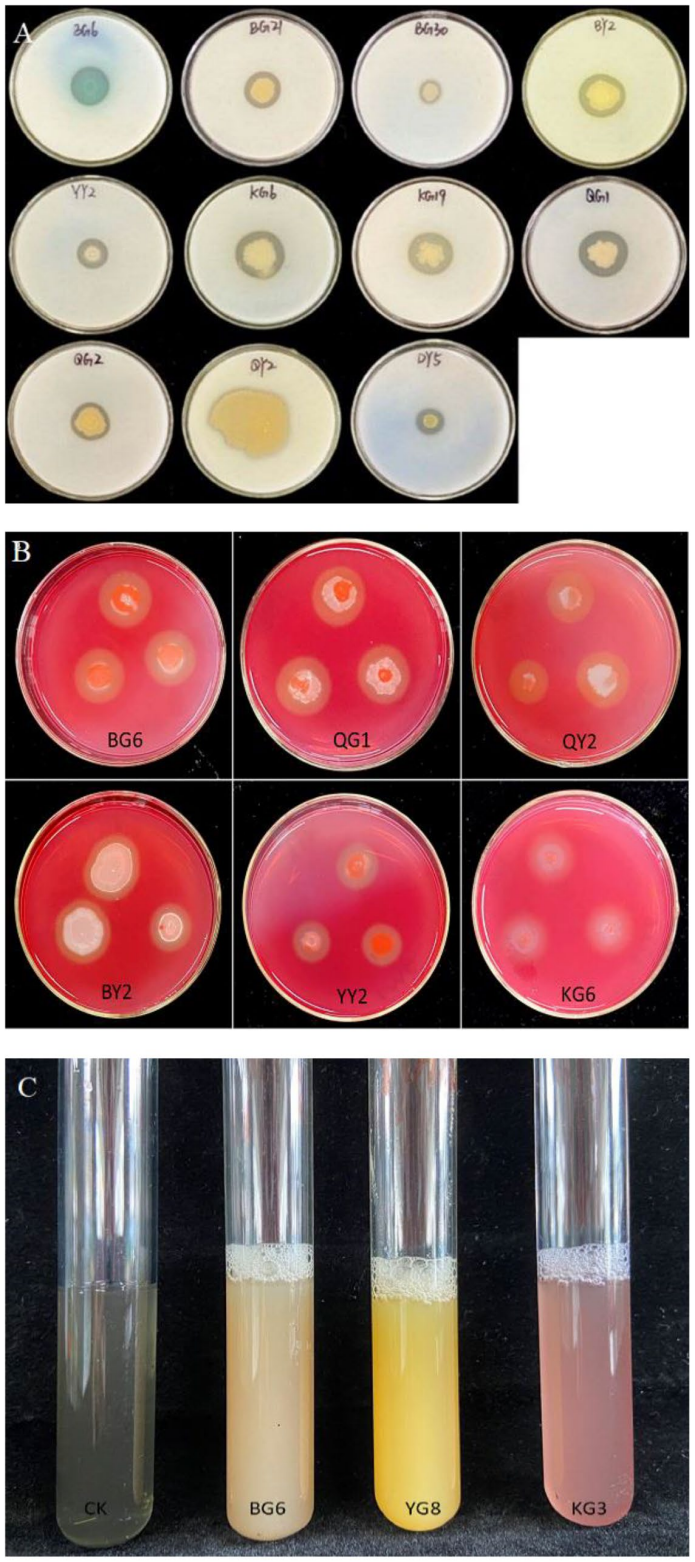
The results of growth-promoting characteristics of related strains: Proteinase production **(A)**, Cellulase production **(B)**, and Siderophore production **(C)**.

The ability of P solubilization of the strains BG6, BG30, BY2, YG8, YY2, QG1, and QY2 was evaluated, with BG6 having the highest P solubilization, i.e., 130.82 mg L^−1.^ similarly, the strains BG6, BG21, BG30, YG8, KG3, and QY2 were able to solubilize potassium, among all the strains YG8 having the highest K solubilization capacity (116.59 mgL^−1.^). Strains BG6, BG21, BG30, BY2, YY2, KG6, KG19, QG1, QG2, QY2, and DY5 were positive for protease production (Figure 2A), while BG6, QG1, QY2, BY2, YY2, and KG6 isolates were positive for cellulase enzyme (Figure 2B). However, strains LG10, BG21, BY2, KG3, KG39, and KG43 were able to produce siderophore (Figure 2C). In addition, enzymes such as Chitinase and ACC deaminase production were also detected (Supplementary Table 3). Bacterial strains BY2, YG8 and KG39 were positive for Chitinase production. Furthermore, strains BY2, KG43, QG2, and DY5 were able to produce ACC deaminase enzyme.

**Figure 3:**
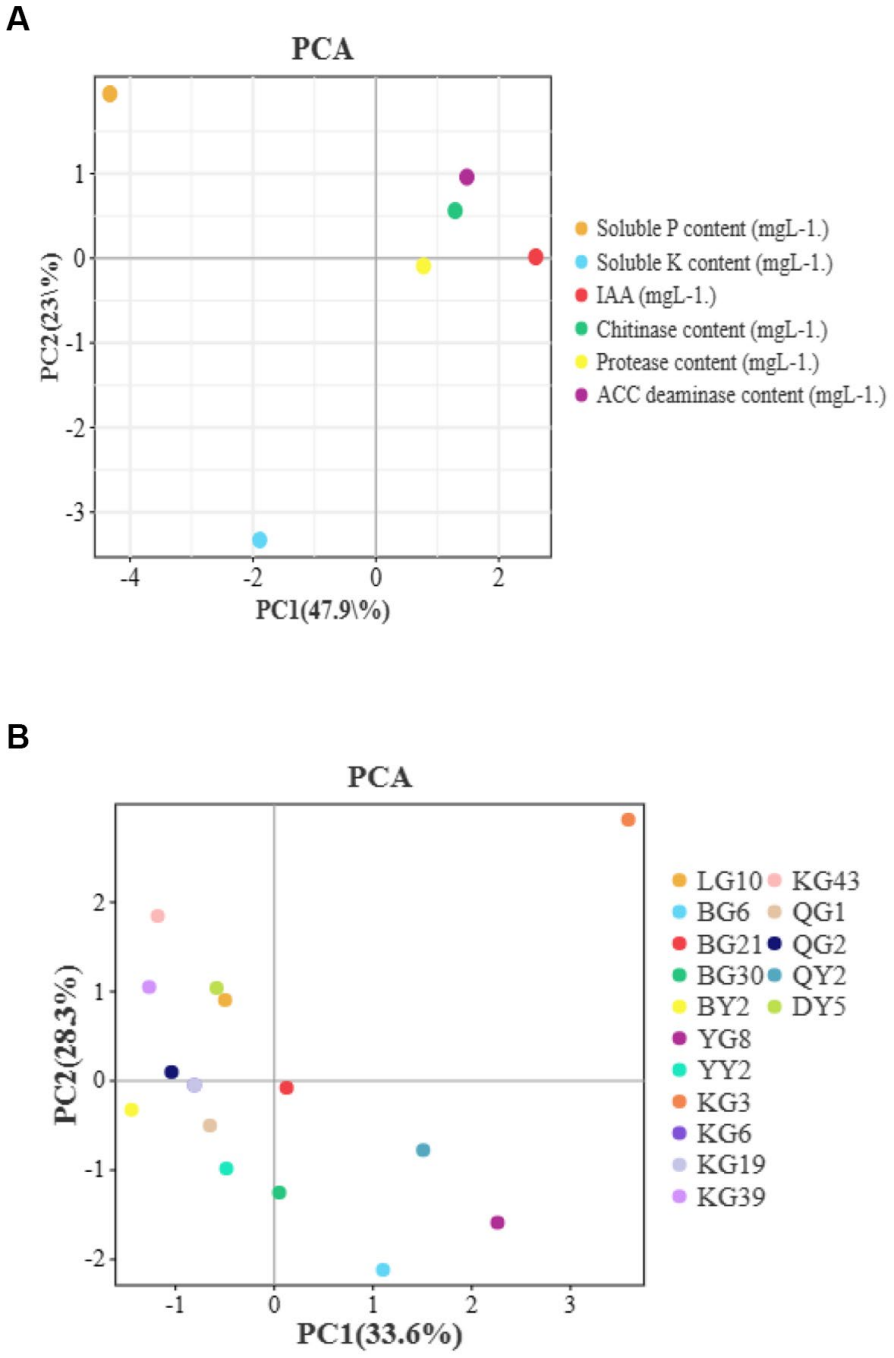
The results of PCA analysis which between different enzymes and plant growth traits **(A)** or isolates **(B)**.

PCA analysis were used to find the relationship between isolates’s different enzymes such as IAA, P solubilization etc. and plant growth (Figure 3). The distance between these points is almost very short. There is a positive relationship among bacterial enzymes and plant growth promotion characteristics.

### Nitrogenase activity assay for the strains

Nitrogen (N) fixation is another plant growth-promoting character (PGP). All the representative isolates were subject to their ability to fix free N. Bacterial isolates BG6, KG3, and KG43 were able to fix N_2_. The highest N fixation rate, i.e., 310.3 nmol-C_2_H_4_/(mLh^−1^) we observed for the strain BG6 and lowest for KG3, i.e., 171.1 nmol-C_2_H_4_/(mLh^−1^).

### Rhizospheric and endophytic bacterial diversity sequencing results and OTU annotations

After quality filter and chimera removal, 2,048,583 effective sequences of 16S rRNA gene region V3-V4 with a length of 400 and 417 bps at 99.9% similarity were obtained from 6 samples. From all the sequencing, 1,501 bacterial OTUs were reported; 363 were common OTUs in 6 samples.

### Bacterial diversity across the samples

**Figure 4:**
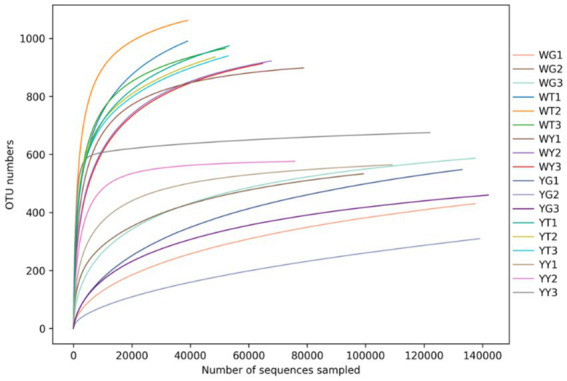
Multi-sample rarefaction curves, each curve represents the species abundance of that sample.

At a 99.9% similarity threshold (Devin et al., 2016), rarefaction analysis assessed OTU richness for a given number of samples based on rarefaction curves (Figure 4). The samples’ curves showed a similar pattern, flattening on the right, indicating that a reasonable number of reads were obtained.

Higher values indicate a more diverse and richly populated community, the alpha diversity index is shown in Table 5. Community diversity is measured by the Chao1, ACE, Shannon, and Simpson indices. The Chao1 index values in the soil community of the rhizosphere were 2.54 and 2.39 times greater than those in the communities of the leaves and roots. The ACE index values of the soil communities in the rhizosphere were 1.36 and 1.60 times higher than those of the leaf and root communities. For Shannon and Simpson diversity indices, rhizosphere soils and leaves performed better than roots. The research indicated that the most numerous and diverse microbial population, followed by leaves and roots, was present in the rhizosphere soil.

**Table 5:** Statistical results of bacterial alpha-diversity in each sample (*p* < 0.05, ANOVA, Tukey-HSD test).

	ACE	Chao1	Simpson	Shannon
YG	995.46	1027.53	0.62	2.06
WG	828.02	842.61	0.83	3.89
YY	1051.95	1062.88	0.97	7.49
WY	1087.29	1086.50	0.98	7.35
YT	1191.36	1184.91	0.99	8.24
WT	1243.73	1259.75	1.00	8.92

### Abundance and taxonomy of the bacterial community

A total of 28 phyla and 548 genera are identified when the bacterial community composition of soil samples from the roots, leaves, and rhizosphere is counted and collated at various classification levels.

**Figure 5:**
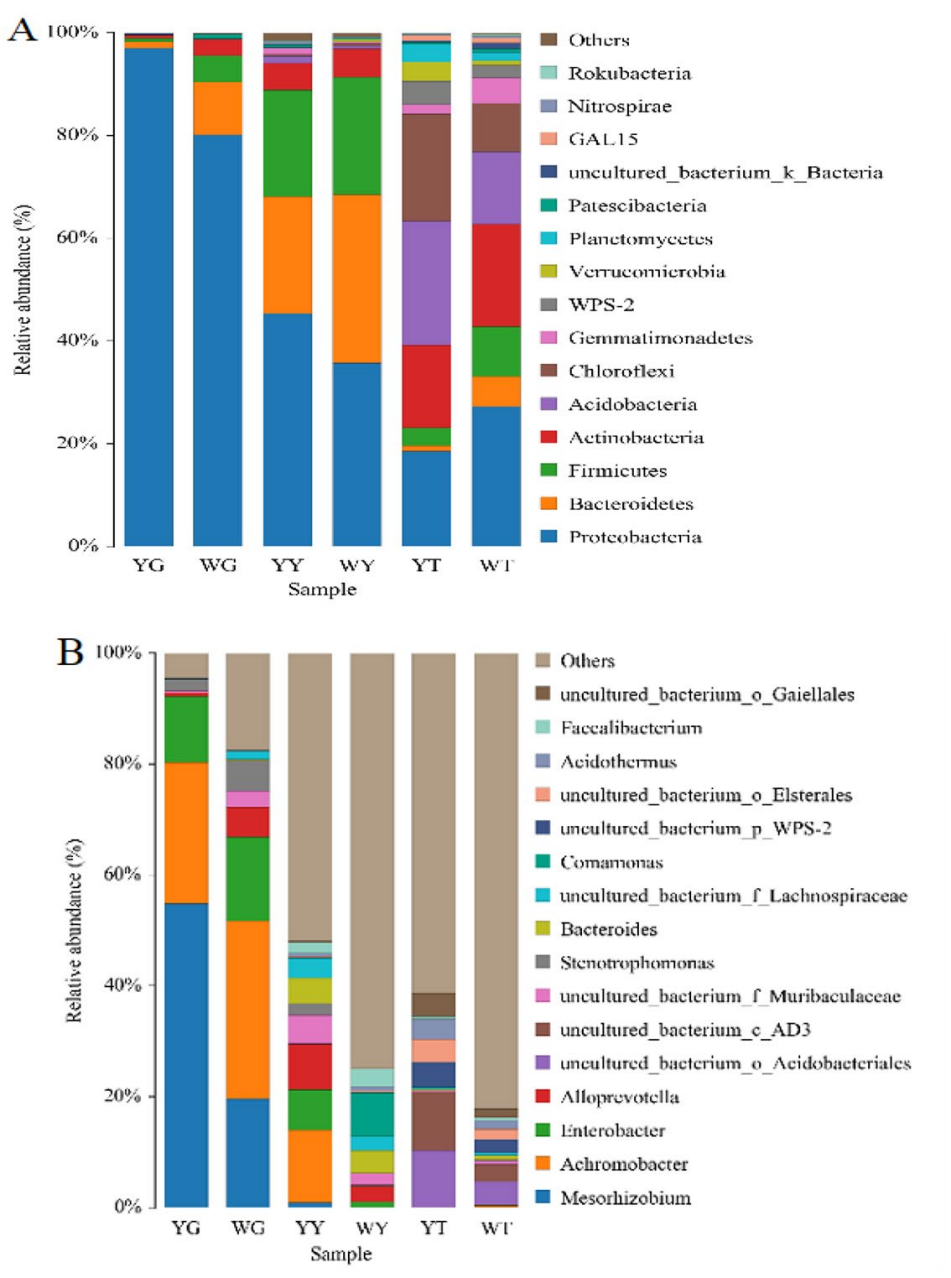
Relative abundances of bacterial community at the phylum level **(A)** and at the genus level **(B)**.

The dominant populations of relative abundance at the phylum- and genus-level for the different samples are given in Figures 4, 5. *Proteobacteria*, *Acidobacteria*, *Actinobacteria*, *Chloroflexi*, and *Firmicutes* were the five groups most common in the rhizosphere soil, making up 22.58, 19.48, 17.82, 15.53, and 6.45%, respectively. In the sample YT, the relative abundance of *Actinobacteria* and *Firmicutes* was lower than that of the WT, and the relative abundance of *Proteobacteri*a, *Acidobacteria*, and *Chloroflexi* was higher than that of the WT. *Proteobacteria*, *Bacteroide*s, and *Firmicutes* were the most common phyla in leaf and root endophytes, which made up 41.65, 26.75, and 21.46% in the leaves and 89.01, 59.00, and 2.74% in the roots, respectively. In contrast to the sample WG, the sample YG had a greater relative abundance of proteobacteria but a lower relative abundance of *Bacteroidetes* and *Firmicutes*. Compared to sample WY, sample YY had a higher relative abundance of *Proteobacteria* but lower relative abundances of *Bacteroidetes* and Firmicutes.

In all samples, 548 bacterial genera were identified. Twenty-one bacterial genera above 1.00%, or 50.82%, were identified in the rhizosphere soil samples; 15 were non-culturable. Six genera in the root system and 16 bacteria in the leaves are more numerous than 1% in the endogenous bacterial community. The five genera, *Mesorhizobium* (38.10%), *Achromobacteria* (28.61%), Enterobacter (13.43%), *Stenotrophomonas* (3.70%), and *Alloprevotella* (2.91%) were in the root. *Achromobacter* (7.71%) was the most common in the leaf sample.

### Bacterial community structure

**Figure 6:**
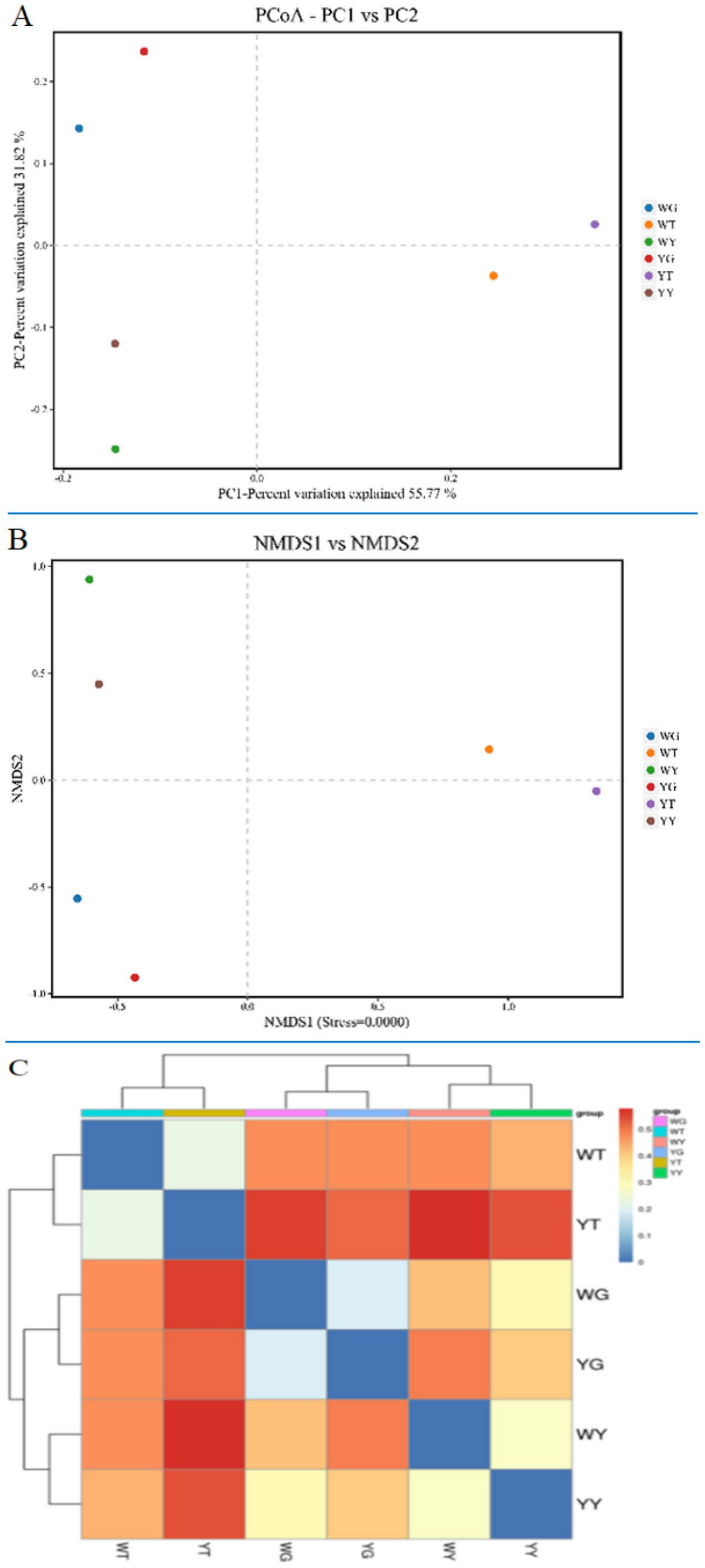
PCoA **(A)** and NMDS **(B)** analysis of bacterial communities and heatmap **(C)** of similarities between the rhizosphere soil bacterial communities and the endophytic bacterial communities of *R. pseudoacacia* ‘Hongsen’.

By using Non-metric Multidimensional Scaling (NMDS) analyses (Gao et al., 2017) based on UniFrac similarity distances and weighted UniFrac-based PCoA, differences in the composition of the bacterial community were identified (Figures 6A,B). The level of similarity increases with sample proximity on the co-ordinate map. The endogenous bacterial communities of the leaf and the root are separated along the second axis, the endogenous bacterial communities of the leaf and the rhizosphere soil are separated along the first axis, and PCoA shows the cumulative contribution rates of the two principal components to be 55.77, 31.82, and 87.59%, respectively.

The bacterial communities of roots and leaves are more similar than those of samples YT and WT, YG and WG. However, YT and WT were different in terms of bacterial community than YY and WY. Based on the weighted UniFrac distance between the clustering heatmap (Figure 6C), the similarity of each sample is reflected by color, and the similarity from blue to red is gradually reduced. It shows how similarity increases among different samples, with roots and leaves showing the highest similarity.

### The difference in the bacterial community across the samples

For statistical significance in abundances of different phyla across the samples (*p* ≤ 0.05) was used. The relative abundance (>0.1%) of *Verrucomicrobia*, *Acidobacteria*, and *Chloro* were significantly (*p* < 0.01) different between YT and WT. In the sample, YY and WY, the *Verrucomicrobia*, *Proteobacteria*, *Firmicutes*, and *Tenericutes* phyla were significantly different, and the relative abundance of these phyla were 0.1% high in the sample YG and WG. About 50 genera in the sample (YT, WT), and 77 genera in (YY, WY) were significantly different from other samples in abundance.

*Mesorhizobium, Achromobacter, and Enterobacter* was the most abundant genera in the root samples. In the samples YG and WG, their relative abundances of *Mesorhizobium* and *Achromobacter* were 55.49 and 21.78%, 19.64 and 31%, respectively (Figure 5).

### Bacterial community function prediction

**Figure 7:**
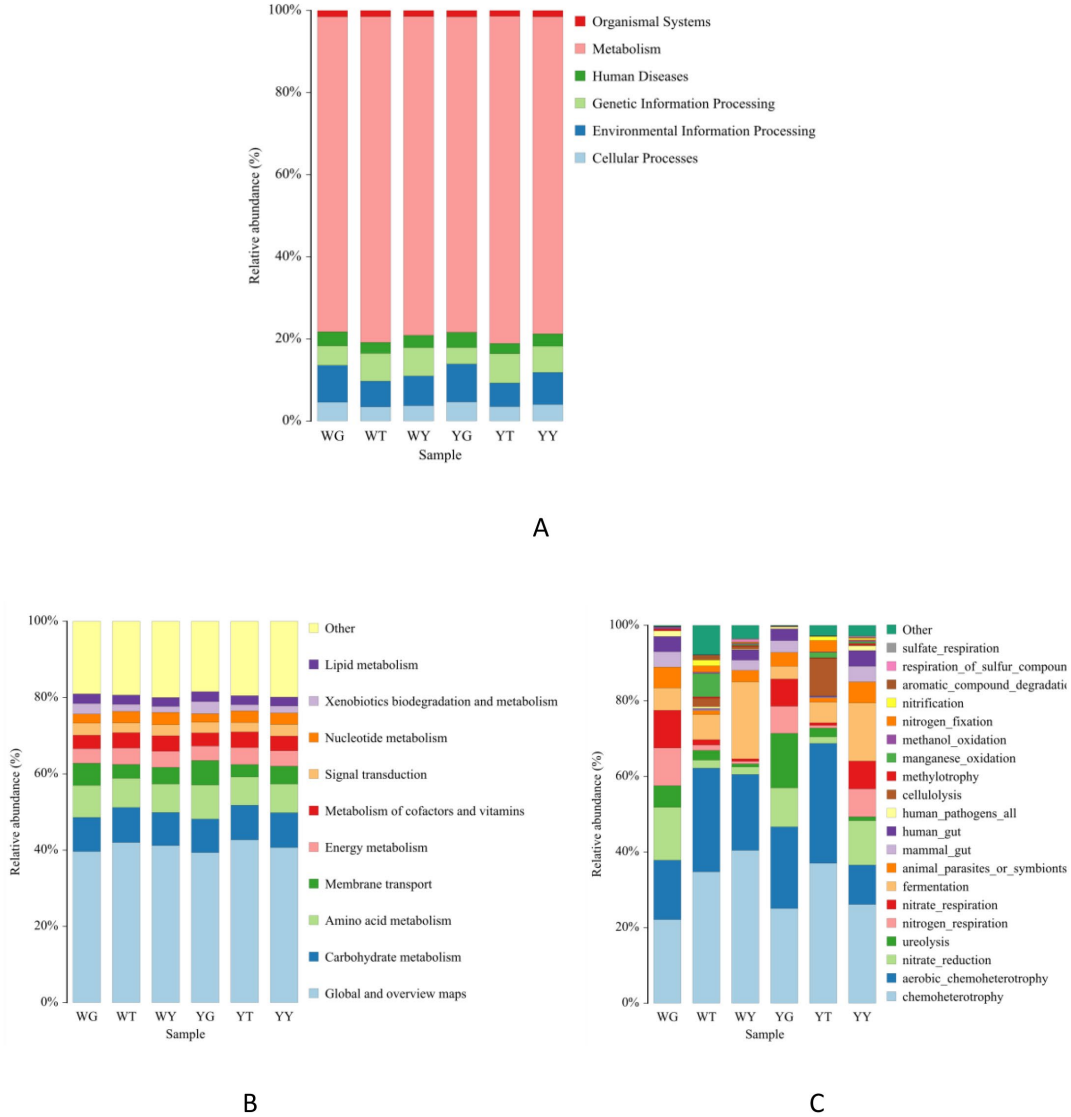
Bacterial community functions predicted (level 1) **(A)** and (level 2) **(B)** by PICRUSt2 and functional ecological diversity of the bacterial community of each sample based on FAPROTAX tool **(C)**.

Figures 7A,B show the prediction function of bacterial communities in the endophytic and rhizosphere. The main dominated functions were categorized into six; these are metabolism (77.92%), environmental information processing (7.55%), genetic information processing (5.99%), cellular processes (4.01%), human diseases (3.16%), and organismal systems (1.47%).

However, we predicted that 44 metabolic pathways in which 14 were the most abundant. These were related to carbohydrates, amino acids, membrane transport, energy, cofactors and vitamins, nucleotide, signal transduction, lipid, and exobiodegradation metabolism. The rhizosphere and endosphere bacterial communities of *R. pseudoacacia* possess diverse functions. Therefore, it is suggested to use endophytes in future rhizospheric engineering.

After functional annotating of soil bacterial communities using FAPROTAX, 53 functional population groupings were discovered (Figure 7C). About 20 functional categories among them, which were relatively abundant, composed 97.21% of the total abundance. The highest abundance, about 10%, was predicted for chemoheterotroph and aerobic chemoheterotroph communities in these 20 functional groups. In addition, other abundant functional groups were related to nitrate reduction, ureolysis, nitrogen and nitrate respiration, and fermentation.

The relative abundance of nitrate reduction, nitrogen respiration, chemoheterotroph, and aerobic chemoheterotroph populations was higher in the rhizosphere as compared to the endosphere. On the other hand, populations like ureolysis, methylotrophy, and fermenters have a higher relative abundance in rhizosphere soil samples than leaf samples.

## Discussions

### *Robinia pseudoacacia* ‘Hongsen’ possess diverse endophytes

In this research, 210 endophytic bacteria were isolated from the roots, root nodules, and leaves of *R. pseudoacacia* ‘Hongsen.’138 strains were from roots and root nodules, accounting for 65.71% of the isolates. Furthermore, 72 strains were isolated from leaves, making up 34.29% of the isolated endophytes. A recent study isolated 28 entophytic bacteria from the leaves and roots of *Cenchrus fungigraminus* (Jing et al., 2022). Similarly, 24 endophytes were isolated from different parts of *Bruguiera gymnorhiza* (Mahmud et al., 2017). However, the number of endophytic bacteria in any plant species is very high, never so far isolated more than 200 bacterial species. For example, a study only isolated 120 strains from *Zea may* (Marag and Suman, 2018; Abedinzadeh et al., 2019). The current study reported more isolates with great biotechnological and agricultural potential than the above studies.

The total isolated 210 strains can be grouped into 16 groups based on IS-PCR. Each group’s representative strains are divided into eight different bacterial genera. Out of 16 representative strains, eight bacterial strains were identified as *Bacillus*, making up 50% of the total isolates, i.e., 101. *Bacillus* has emerged as the most studied and utilized group of plant endophytes. In addition to supporting host plant growth, stress resistance, and other functions, it also plays a significant role in crop biocontrol and antibiotic production (Gond et al., 2015).

### Functional diversity of *Robinia pseudoacacia* (Hongsen) endophytic bacteria

Endophytes identified and purified from different plant parts in this research had many biological functions. The 16 representative strains possess the P and K solubilization, protease, cellulase, siderophore, and IAA production. Due to their functional diversity, these strains can be used in agriculture research as PGPR or biofertilizers and for the remediation of heavy metals. For example, the production of camphor oil and stain resistance are connected to the colonization and growth of sporogenic bacteria with solid stress resistance (Dasgupta et al., 2020). Additionally, an endophytic bacterium at a fir tree’s root can effectively inhibit pathogenic bacteria’s growth (Morajkar et al., 2019). Similarly, the study of the nodulation mechanism of legumes is made easier due to endophytic bacteria-related research (Duangpaeng et al., 2012; Khan et al., 2020). Furthermore, endophytes can help in colonization of beneficial microbes to the host plant. This contributes as microbial germplasm resources, bio-fertilizers, and biopesticides (Bibi et al., 2018).

### 16S *rRNA* gene sequencing and diversity of endosphere and rhizosphere

The rhizosphere soil and endophytic bacteria in the roots and leaves were sequenced using high-throughput sequencing technology, and the bacterial community structure, composition, and diversity were studied.

All samples contained a total of 543 bacterial genera and 28 bacterial phyla. Like most forests, tea plants, farms, and grasslands, rhizosphere soils are dominated by *Proteobacteria*, *Acidobacteria*, *Actinobacteria*, *Chloroflexi*, and *Firmicutes*. Leaf and root endophyte communities are dominated by *Proteobacteria* (Müller et al., 2016). The phyla *Bacteroidete* and *Firmicutes* are similar to most plants (Bai et al., 2015). Root was dominated by *Mesorhizobium*, *Achromobacter*, *Enterobacter*, *Stenotrophomonas*, and *Alloprevotella* genera. However, shoots dominant genera were while *Achromobacter*, *Alloprevotella*, and *Enterobacter*. Similar results were also reported by previous studies (Miri et al., 2019; Yuan et al., 2020). Since leaves and roots, rhizosphere soils have the highest bacterial community richness and diversity, according to an alpha diversity analysis. In the same tissues of nodular plants and rhizomes, beta diversity analysis showed no significant differences in endophytes and rhizosphere soil bacteria. However, there were significant differences in the bacterial communities of different tissues and rhizosphere soils. Endophytic bacteria are primarily obtained from soil, but they also have their particular habitat types. The root with nodules, have the dominant genus *Mesorhizobium* with more than 50% abundance. This shows the possible role of *Mesorhizobium* in rhizomes formation in the host plant. The primary root nodules formed at the roots of *R. pseudoacacia* ‘Hongsen’ are medium slow rhizobia; based on 16S *rRNA* analysis of endosphere and rhizosphere, there were significant differences in the bacterial communities of roots, leaves, and rhizospheres, soil bacterial richness was higher than that of endophytic bacteria. The community structure of roots and leaves was more similar compared to the rhizosphere. Endophytic bacteria with and without nodule roots differed significantly, and it was hypothesized that the function of endophytic bacteria of *R. pseudoacacia* ‘Hongsen’ mainly was related to the metabolic process such as carbonhydrate metabolism, amino acid metabolism, energy metabolism, etc.

### Functional diversity of the bacterial community

In the present study, the biological functions of endophytes were diverse. Representative strain BG6 (*Pseudomonas aeruginosa*) can dissolve phosphorus, potassium, protease, and cellulase. A study isolated *P. aeruginosa* from *Carthamus tinctorious* L. has a phosphorous solubilizing function (Zhang et al., 2016). Similarly, *P. aeruginosa* isolated from eutrophication water in a cold area has a solubilizing effect on Ca_3_(PO_4_)_2_ and AlPO_4_ (Banerjee et al., 2010). Isolate DY5 (*Microbacterium paraoxydans*) has the highest protease production. The study has confirmed that this strain isolated from licorice has antagonistic activity against *the Fusarium* leaf spot of grass fruit, *Petopotiopsis pseudostem* black spot of grass fruit, and other pathogens (Wang et al., 2019). However, strain KG3 (*Pseudomonas mosselii*) can produce IAA. *P. mosselii* is related to better growth and height of the plant (Wang et al., 2006).

Endophyte BG21 (*Priestia aryabhattai*) has the functions of potassium hydrolysis, protease production, and IAA production. In addition, it can potentially degrade benzoate and antagonize fungal and bacterial plant pathogens (Esikova et al., 2021). BG30 (*Bacillus zhangzhouensis*) functions as P solubilizing and removing potassium. A study has confirmed that it has antagonistic effects on the pathogens of rice bacterial leaf streak and bacterial blight of rice (Wu et al., 2015). Bacterial isolate QY2 (*Bacillus pumilus*) can solubilize phosphorus, solve potassium, and produce protease and cellulase. In *Oryza sativa* L., this strain can promote growth and root indexes (Ouyabe et al., 2020). These strains with different functions can be used for further research and application in sustainable agriculture.

### Soil process and metabolism of the bacterial community

Our results showed that the microbial communities have cellulose, nitrate, nitrification, and fermentation related enzymes. This shows that the rhizosphere is the hot spot for most soil and plant processes. The endophytes affect rhizosphere chemistry. For example it has changed the root exudates which significantly increased the C and N fraction of the rhizosphere (Guo et al., 2015). Mikola et al. (2016) tested whether the infection of meadow fescue, *Schedonorus pratensis*, by *Epichloë uncinata* can decelerate litter decomposition, N release, increase soil C and N accumulation and lower the availability of mineral N in the soil under treatment. They found that *E. uncinata* infection neither affected meadow fescue litter N%, mass loss, nor N release. Soil C and N content and NH_4_ and NO_3_ contents did not differ between the endophyte-infected and non-infected grass plots, and litter did not decompose faster when endophytes were used. It suggests that endophytes may not decrease the benefit of the endophyte-grass symbiosis by reducing soil fertility.

Soto-Barajas et al. (2016), using several *Epichloë* endophytes and *Lolium perenne* showed endophyte-infected plants had significantly lower P, Ca, S, B, fiber, and lignin contents and higher Mn absorption than non-inoculated plants. Slaughter et al. (2016) showed that endophytic strains have different nitrogen utilization. All the studies which mentioned above clearly showed the soil process and metabolism of the bacterial community on the benefits exerted by endophytic bacteria and fungi on plants. In this research, traditional methods and high-throughput analysis which were same as previous studies obtained the prediction of soil bacterial community function. The results showed that it was closely related to the metabolic process of *R. pseudoacacia* ‘Hongsen’ and metabolic processes such as carbonhydrate metabolism, amino acid metabolism, energy metabolism, were involved.

## Conclusion

Two hundred ten bacterial endophyteswere isolated from *R. pseudoacacia* ‘Hongsen’, indicating a diverse bacterial community. These isolates have great potential to perform various functions. Functional prediction analysis revealed that their functions are related to metabolism. In the future, these isolated strains can be used in different agriculture-related studies. We suggest exploring the endophytes of other plant species as they have great potential to be used in stress biology.

## Data availability statement

The datasets presented in this study are deposited in the NCBI repository, under accession number PRJNA999018: https://www.ncbi.nlm.nih.gov/bioproject/PRJNA999018.

## Author contributions

GP, ZT, and MH conceived the study. MH and JMa designed and conducted the experiments and wrote the manuscript. LC, JMo, LH, and QL contributed to the data analyze. GP and ZT guided the experiment. All authors agreed to be accountable for the content of the work.

## Funding

This work was supported by the National Natural Science Foundation of China (31970001) and Science and Technology Project of Guangdong Province (2018A050506075).

## Conflict of interest

The authors declare that the research was conducted in the absence of any commercial or financial relationships that could be construed as a potential conflict of interest.

## Publisher’s note

All claims expressed in this article are solely those of the authors and do not necessarily represent those of their affiliated organizations, or those of the publisher, the editors and the reviewers. Any product that may be evaluated in this article, or claim that may be made by its manufacturer, is not guaranteed or endorsed by the publisher.
