# Supplementary material for: Biological functions of endophytic bacteria in Robinia pseudoacacia ‘Hongsen’
Source: Front Microbiol. 2023 Aug 9;14:1128727. doi: 10.3389/fmicb.2023.1128727 (PMC10446884; doi:10.3389/fmicb.2023.1128727)
Supplement: Supplementary file 1 [file Table_1.docx]

SUPPLEMENT TABLE 1 Endophytic strains isolated from the plant tissues of *R. pseudoacacia* 'Hongsen'

| Strain number | Source | Culture medium |
| --- | --- | --- |
| LG1~24 | Roots and root nodules | LB |
| LY1~14 | Leaves | LB |
| BG1~33 | Roots and root nodules | BPA |
| BY1~14 | Leaves | BPA |
| YG1~9 | Roots and root nodules | YPD |
| YY1~6 | Leaves | YPD |
| KG1~44 | Roots and root nodules | KMB |
| KY1~5 | Leaves | KMB |
| QG1~16 | Roots and root nodules | NA |
| QY1~17 | Leaves | NA |
| DG1~12 | Roots and root nodules | DN |
| DY1~16 | Leaves | DN |
