# Supplementary material for: Biological functions of endophytic bacteria in Robinia pseudoacacia ‘Hongsen’
Source: Front Microbiol. 2023 Aug 9;14:1128727. doi: 10.3389/fmicb.2023.1128727 (PMC10446884; doi:10.3389/fmicb.2023.1128727)
Supplement: Supplementary file 2 [file Table_2.docx]

SUPPLEMENT TABLE 2 Growth-promoting characteristics of strains

| Strain number | Chitinase  content (mgL^-1.^) | Protease content (mgL^-1.^) | ACC deaminase content (mgL^-1.^) |
| --- | --- | --- | --- |
| LG10 | 0 | 0 | 0 |
| BG6 | 0 | 0 | 0 |
| BG21 | 0 | 0 | 0 |
| BG30 | 0 | 0 | 0 |
| BY2 | 7.59 | 0 | 16.80 |
| YG8 | 3.51 | 8.68 | 0 |
| YY2 | 0 | 0 | 0 |
| KG3 | 0 | 21.27 | 0 |
| KG6 | 0 | 0 | 0 |
| KG19 | 0 | 0 | 0 |
| KG39 | 21.78 | 0 | 0 |
| KG43 | 0 | 0 | 15.6 |
| QG1 | 0 | 0 | 0 |
| QG2 | 0 | 0 | 4.41 |
| QY2 | 0 | 7.49 | 0 |
| DY5 | 0 | 0 | 2.04 |
