# Supplementary figures and images for: Biological functions of endophytic bacteria in Robinia pseudoacacia ‘Hongsen’
Source: Front Microbiol. 2023 Aug 9;14:1128727. doi: 10.3389/fmicb.2023.1128727 (PMC10446884; doi:10.3389/fmicb.2023.1128727)

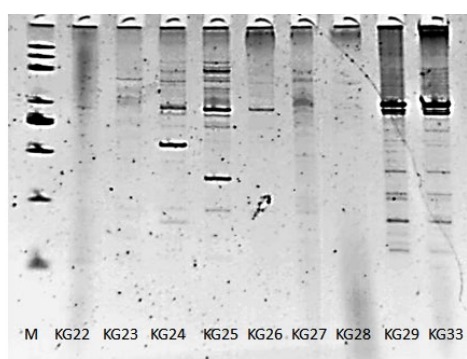

J3-7

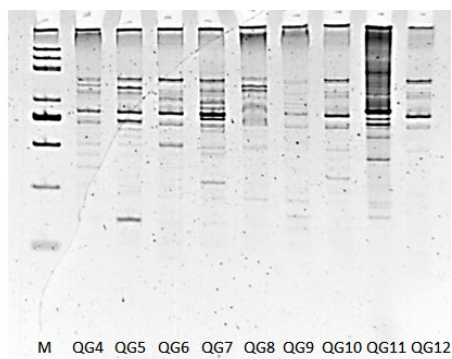

J3-13

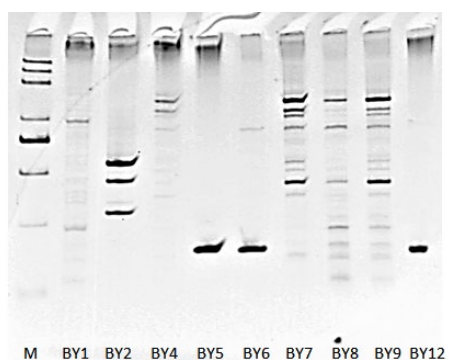

J3-20

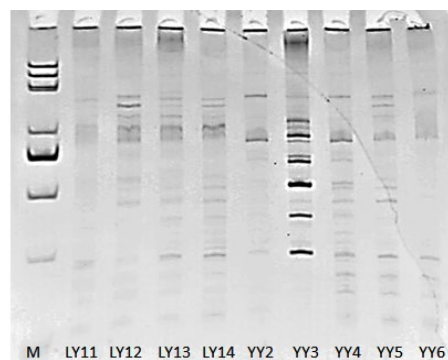

J3-21

SUPPLEMENT FIGURE 1 IS-PCR data showed representative gel results

Supplement: Supplementary file 3 [file Image_1.pdf]

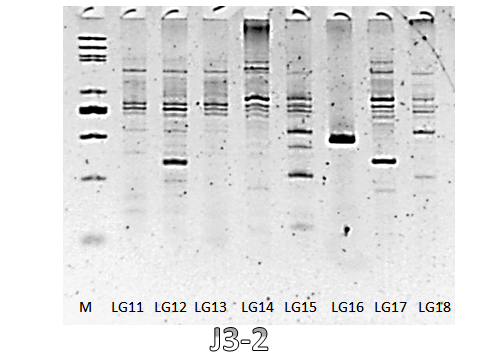

Supplement: Supplementary file 4 [file Image_2.PNG]

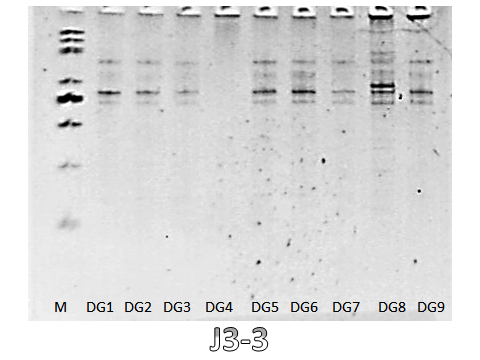

Supplement: Supplementary file 5 [file Image_3.PNG]

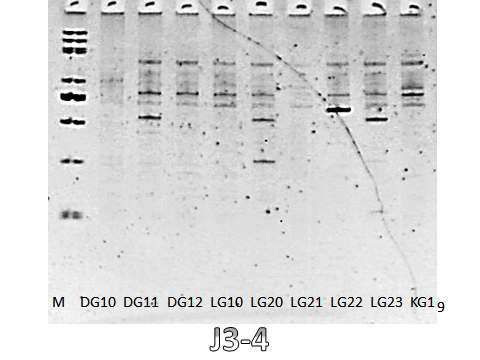

Supplement: Supplementary file 6 [file Image_4.PNG]

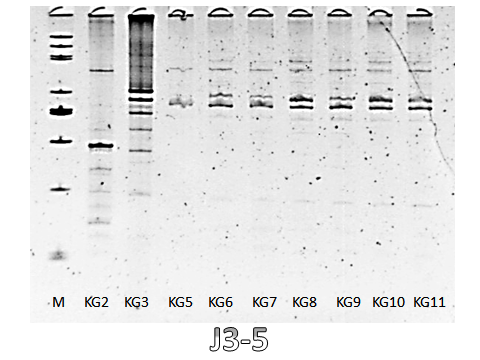

Supplement: Supplementary file 7 [file Image_5.PNG]

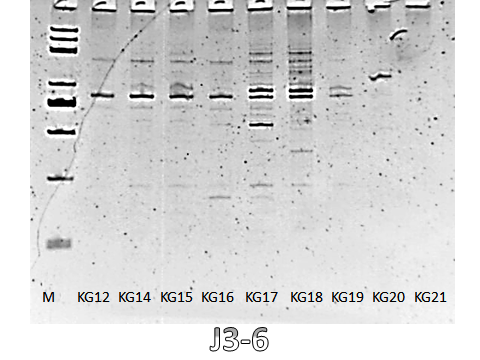

Supplement: Supplementary file 8 [file Image_6.PNG]

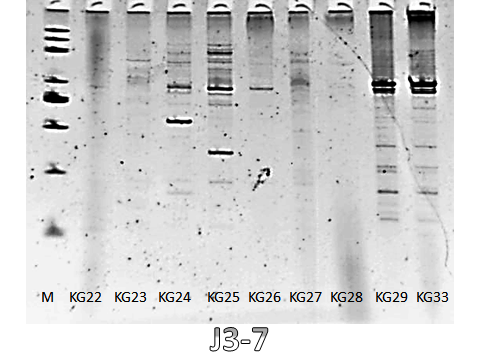

Supplement: Supplementary file 9 [file Image_7.PNG]

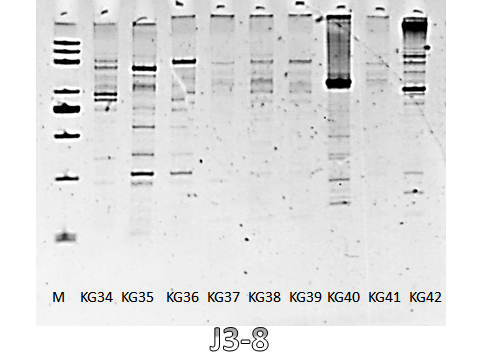

Supplement: Supplementary file 10 [file Image_8.PNG]

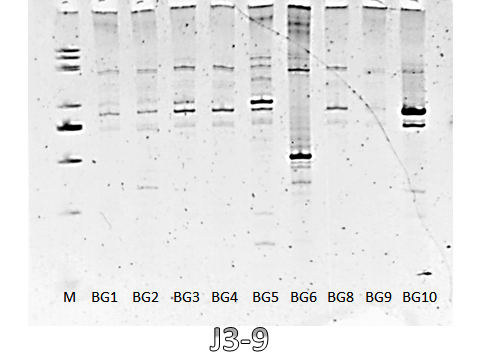

Supplement: Supplementary file 11 [file Image_9.PNG]

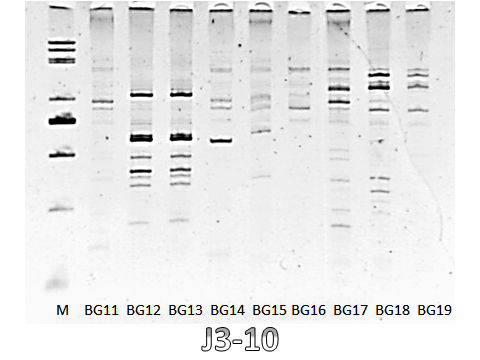

Supplement: Supplementary file 12 [file Image_10.PNG]

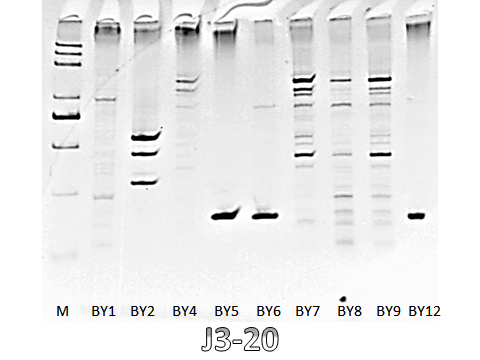

Supplement: Supplementary file 13 [file Image_11.PNG]

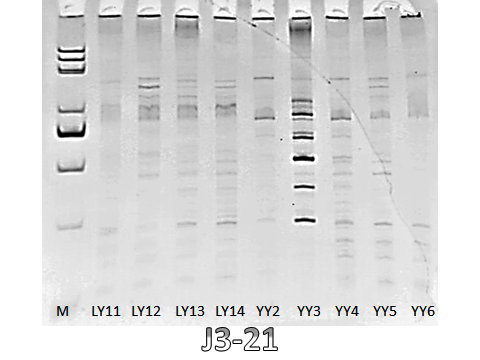

Supplement: Supplementary file 14 [file Image_12.PNG]
